# Single cell transcriptome in aneuploidies reveals mechanisms of gene dosage imbalance

**DOI:** 10.1038/s41467-019-12273-8

**Published:** 2019-10-03

**Authors:** Georgios Stamoulis, Marco Garieri, Periklis Makrythanasis, Audrey Letourneau, Michel Guipponi, Nikolaos Panousis, Frédérique Sloan-Béna, Emilie Falconnet, Pascale Ribaux, Christelle Borel, Federico Santoni, Stylianos E. Antonarakis

**Affiliations:** 1 Department of Genetic Medicine and Development, University of Geneva Medical School, 1211 Geneva 4, Geneva, Switzerland; 2 Geneva University Hospitals, Service of Genetic Medicine, 1211 Geneva 4, Geneva, Switzerland; 3 Service of Endocrinology, Diabetes and Metabolism, University Hospital of Lausanne - CHUV, Lausanne, 1011 Switzerland; 4 iGE3 Institute of Genetics and Genomics of Geneva, University of Geneva, 1211 Geneva 4, Geneva, Switzerland; 5 Present Address: Biomedical Research Institute Academy of Athens, Athens, Greece

**Keywords:** Gene expression, Gene regulation, Diseases

## Abstract

Aneuploidy is a major source of gene dosage imbalance due to copy number alterations (CNA), and viable human trisomies are model disorders of altered gene expression. We study gene and allele-specific expression (ASE) of 9668 single-cell fibroblasts from trisomy 21 (T21) discordant twins and from mosaic T21, T18, T13 and T8. We examine 928 single cells with deep scRNAseq. Expected and observed overexpression of trisomic genes in trisomic vs. diploid bulk RNAseq is not detectable in trisomic vs. diploid single cells. Instead, for trisomic genes with low-to-average expression, their altered gene dosage is mainly due to the higher fraction of trisomic cells simultaneously expressing these genes, in agreement with a stochastic 2-state burst-like model of transcription. These results, confirmed in a further analysis of 8740 single fibroblasts with shallow scRNAseq, suggest that the specific transcriptional profile of each gene contributes to the phenotypic variability of trisomies. We propose an improved model to understand the effects of CNA and, generally, of gene regulation on gene dosage imbalance.

## Abstract (web summary)

Gene dosage anomalies such as those caused by aneuploidy underlie diseases including Down syndrome. Here, the authors perform allele-specific single cell transcriptome analysis to investigate the mechanisms of gene dosage imbalance in fibroblasts with trisomies T21, T18, T13 and T8.

## Introduction

The biochemical processes underlying complex cellular functions rely on a precise and timely dosage of their constitutive elements and, in particular, of protein stoichiometry^1^. Protein production is inherently connected with gene expression level, which, in turn, is regulated by several factors of genetic and epigenetic nature^2^. Perturbation of this equilibrium may induce severe cellular and organismal phenotypes. Genomic copy number alterations (CNA) such as duplications and deletions, result in gene expression imbalance^3^ and is associated with reproducible phenotypes as it is the case in aneuploidies. However the respective functional mechanisms are not well understood.

Aneuploidy is a well-known source of gene dosage imbalance through CNA. In particular trisomies are considered to be disorders of altered gene expression of the majority of genes on the supernumerary chromosomes (gene dosage sensitive genes)^4–8^. Trisomy 21 (T21—Down syndrome) is the most common human aneuploidy compatible with postnatal survival, and has been extensively used as a model to study trisomies^9^. Other common trisomies include Trisomy 18 (T18—Edwards’s syndrome) and Trisomy 13 (T13—Patau syndrome)^10–13^. Phenotypes observed in trisomies have been attributed from bulk RNA-seq studies to the gene dosage imbalance, ~1.5 fold higher for the trisomic genes as compared to their diploid counterparts^6,14–16^. However the causative links between altered gene expression and phenotypes in aneuploidies are not known. To understand the molecular basis of trisomy phenotypes, we explored gene expression profiles in single trisomic cells. Hitherto, single-cell RNA-seq (scRNAseq) studies have revealed pervasive genome-wide skewed monoallelic gene expression in diploid cells^17^, but also variability and gradation of gene expression for different genomic phenomena/processes, such as imprinting^18^ and X-inactivation^19^. These phenomena are likely the outcomes of the discrete and stochastic nature of RNA transcription with each gene bearing his own specific regulation^20,21^. In diploid cells, it has been already shown that the large majority of genes respond to a 2-state (ON-OFF) burst-like model of transcription^22^ and core promoter elements and enhancers regulate transcriptional burst size and frequency, respectively^23^. Moreover the transcriptional kinetics of the two alleles of a gene are uncoupled and the alleles are transcribed in two substantially stochastic^24^ and independent processes^23^. It is still unclear how, in CNA, the presence of additional alleles impacts the transcriptional activity and causes gene dosage imbalance. Here we present the comparative analysis of scRNAseq from trisomic and matched isogenic diploid fibroblasts. We show that burst-like transcription shapes gene dosage imbalance in trisomies. In agreement with the 2-state burst-like model, we provide evidence that, in trisomic cells, the additional allele is independently transcribed, leading to an increased monoallelic expression with respect to the diploid cells, and a significant fraction of trisomic cells simultaneously activating gene expression as compared to diploid controls.

## Results

### Identification of trisomic cells in mosaic cell population

We used six different cell lines of skin fibroblasts from six individuals: two samples are from a pair of monozygotic twins discordant for T21^25^; four were from individuals mosaics for T21: CM05287, T13: GM00503, T18: AG13074, T8: GM02596 (Supplementary Fig. 1 and Supplementary Table 1).

To reduce the allele dropout effect, following the strategy introduced in^26^, we performed a split cell experiment where we manually split the content of a single cell and independently performed cDNA synthesis in separate tubes. After sequencing we focused on the common sites detected in the split cells. Common sites discordant for ASE are a bias introduced by the allele dropout effect. We observed that discordant monoallelic sites driven by allelic dropout almost vanished (<1.5%) at RPSM (Reads Per Site per Million) = 20, similarly to what previously observed^26^ (Supplementary Fig. 2).

In order to classify trisomic and diploid cells in a mosaic trisomy cell population we developed an iterative clustering method based on k-means (k = 2) using two metrics: the average cellular gene expression and ASE at heterozygous sites, both measured from the genes located on the supernumerary chromosome (details in Methods). Briefly, after quality control (doublets removal^19^ - Supplementary Fig. 3) informative heterozygous sites were obtained by whole genome sequencing (WGS) and ASE was calculated considering the most covered heterozygous site per gene. Each site of the triplicated chromosome has the allele combination ABB or BAA with two identical alleles (double allele) and one unique allele. At each round of the iteration, the double allele is predicted for each heterozygous site from the average ASE of each predicted single trisomic cell and the status (diploid or trisomic) of a cell is (re)classified by the k(=2)-means algorithm. Convergence is reached when the status of all cells is stable (Supplementary Fig. 4). We examined the accuracy of this method with a test-set of 316 diploid and trisomic single cells derived from a pair of monozygotic twins discordant for T21. We assigned the correct cellular status with an accuracy of ~95% (5-fold cross validation). It is not currently possible to independently validate the individual calls of the cells that have already been sequenced. However, as a further support of the reliability of the algorithm, the estimated proportion of cells in the different mosaic cell lines (mosaic T21, T8, T13, T18) is concordant with the degree of mosaicism derived by fluorescence in situ hybridization (FISH) (Supplementary Fig. 4). These results show that the single-cell ASE analysis in combination with the average cellular expression for triplicated genes can be used to computationally classify trisomic and diploid cells in samples from mosaic individuals.

### Monoallelic gene expression in trisomic single cell

**Fig. 1:**
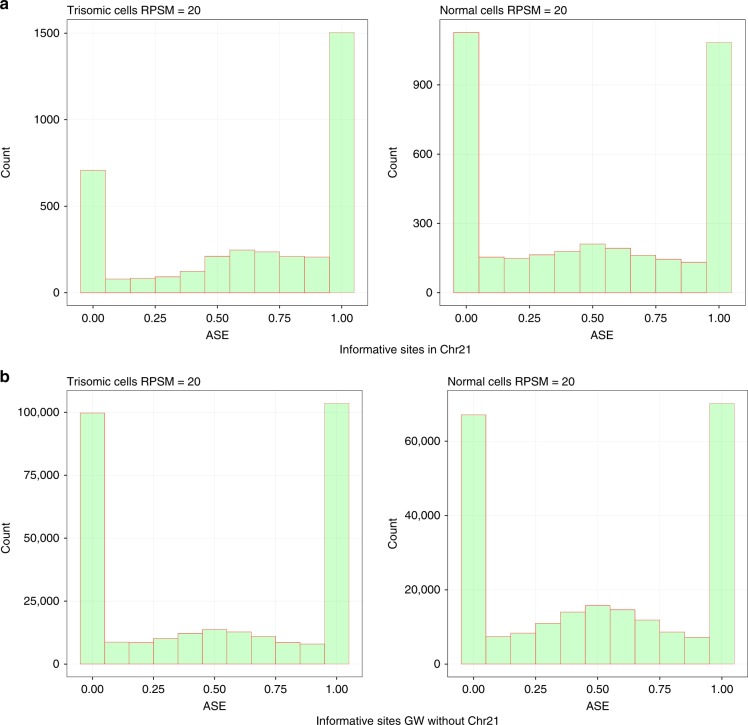
Histogram of SC ASE observations in Monozygotic Twins Discordant for DS. **a** Histogram of ASE observations in chr21 in single cells. Similarly to genome-wide observations, monoallelic ASE in chr21 is prevalent (46.5% diploid – 59.39% trisomic. Notably, for trisomic SC, monoallelic ASEs on chr21 of the double allele (0.9-1) are twice as many of monoallelic ASEs of the single allele (0–0.1). Moreover biallelic observations in trisomic single cells are not centered at 0.5 as in diploid single cells, but at 0.66 (2/3). **a** Histogram of genome-wide ASE observations in SC excluding chr21 (Blue = diploid Twin, Red = trisomic Twin). High prevalence of monoallelic ASE observations was observed for both groups (60.14% diploid – 70.3% trisomic). Source data are provided in the public repository

**Fig. 2:**
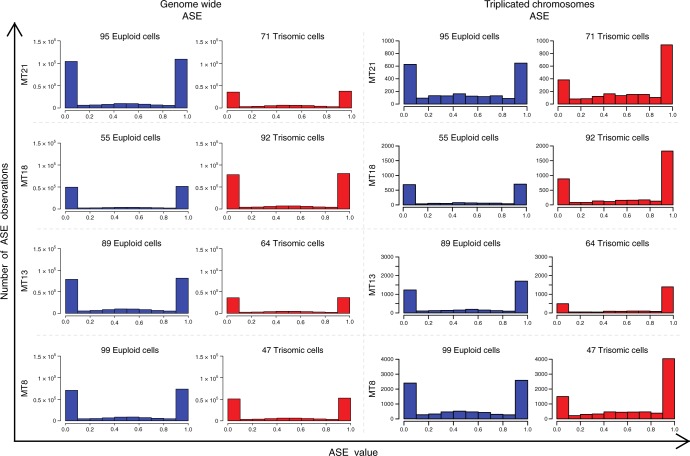
Histogram of SC ASE observations in individuals mosaic for different trisomies. Each row represents one mosaic individual. Left Panel: Histogram of genome-wide ASE observations in SC excluding supernumerary chromosomes. High prevalence of monoallelic ASE observations was observed in all groups. Right panel: Histogram of ASE observations in SC in the supernumerary chromosomes. In all supernumerary chromosomes monoallelic ASE observations represent again the higher fraction of ASE observations, similarly to Genome Wide observations. In the trisomic group, monoallelic observations on supernumerary chromosomes for double allele (0.9–1) are proportionally higher than monoallelic observations of the single allele (0-0.1). Moreover the distribution of the biallelic observations in trisomic single cells is not centered as in diploid single cells, but shifted towards the double allele. (Blue = diploid, Red = trisomic). MT = Mosaic Trisomy. Source data are provided in the public repository

Previous studies on allelic expression in diploid cell population have reported pervasive random skewed monoallelic gene expression at the single-cell level^17,27,28^, i.e., cells expressed predominantly one allele (A or B) at a given time. We observed the same phenomenon in our normal and trisomic cell populations with around 20–22% of genes presenting a monoallelic expression (ASE ≤ 0.1; ASE ≥ 0.9, average among all sites within a gene), in line with what previously reported^26^. In the diploid fraction of the genome, in twins’ fibroblasts discordant for T21, 60.1% of the heterozygous sites showed monoallelic coverage (ASE ≤ 0.1; ASE ≥ 0.9) in the diploid cells and 70.3% in T21 cells (Fig. 1). Similar results were obtained for the diploid fraction of the genome for mosaic T21 cells and the other mosaic trisomies T8, T18, and T13 cells (Fig. 2). In the monozygotic twins discordant for T21, the fraction of monoallelic ASE observations from chromosome 21 sites in diploid cells was 46.5% and 59.4% in T21 cells (Fig. 1). In agreement with random selection of the transcribed allele, the fraction of trisomic informative sites exclusively expressing the unique allele (0 ≤ ASE ≤ 0.1) was close to 1/3 of the total monoallelic observations. Accordingly, in all trisomy samples, the double alleles on the supernumerary chromosome were detected almost 2 times more frequently than the unique alleles (Fig. 2). Moreover, the mean of the distribution of biallelic observations (0.1 < ASE < 0.9) in trisomic single cells was not equal to 0.5 as in diploid single cells, but shifted towards 0.66 (Figs. 1 and 2). Importantly, these observations are not dependent on the chosen RPSM threshold (Supplementary Fig. 5). These observations support a stochastic model of allelic selection by the transcriptional machinery where the probability of an allele to be expressed is linearly dependent on its respective copy number.

### Monoallelic expression correlates with expression level

**Fig. 3:**
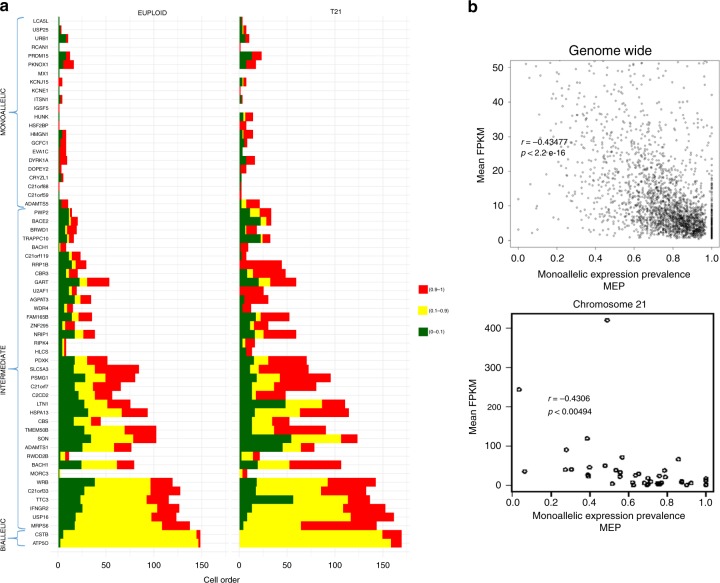
Prevalence of monoallelic expression in supernumerary chromosomes. **a** Classification of genes in three groups (Monoallelic, Intermediate, and Biallelic). Genes for which >80% of cells show monoallelic expression were classified as monoallelic (ASE ≤ 0.1 or ASE ≥ 0.9); genes with 20–80% of cells with 0.1 ≤ ASE ≤ 0.9 were classified as intermediate; genes with <20% of cells with 0.1 ≤ ASE ≤0.9 were classified as biallelic. **b** Monoallelic prevalence is negatively correlated with level of gene expression both genome-wide (diploid fraction of the genome—upper panel) and within the trisomic fraction of the genome (chr21—lower panel). Source data are provided as Source Data file

To further investigate at the single-cell level the gene dosage effect in trisomic fibroblasts, we classified the triplicated genes based on their monoallelic expression prevalence (MEP). MEP represents the fraction of cells per gene with ASE ≤ 0.1 or ASE ≥ 0.9 at the heterozygous site with the highest number of cellular ASE observations (Supplementary Fig. 6). We classified the triplicated genes in three groups: (i) Monoallelic genes: MEP > 80% in diploid and trisomic cells; (ii) Intermediate genes with MEP between 20% and 80%; and (iii) Biallelic genes with MEP < 20% (Fig. 3a). Out of a total of 390 genes, 32% were classified as monoallelic, 66% as intermediate and only 2% as biallelic (Fig. 3a, Supplementary Fig. 7). Overall this classification was concordant for diploid and trisomic cells and consistent with previous single-cell RNA-seq study^26^.

We observed a significant negative correlation (spearman ρ = −0.43, *p* = 5e-3) between ASE and the level of gene expression of the corresponding triplicated gene (Fig. 3b, Supplementary Fig. 7). A similar negative correlation (spearman ρ = −0.43, *p* < 2.2e-16) was also observed for diploid genes in both the diploid and the trisomic cells (Fig. 3b). According to the transcriptional bursting model of gene expression, highly expressed genes have short interburst periods and higher burst size (number of transcripts produced per burst) than low expressed genes^29^. Consequently it is frequent to observe in single cells random simultaneous (i.e., biallelic) transcription from the two alleles of highly expressed genes at a given time point. Conversely, low expressed genes have a low transcriptional bursting frequency and therefore the event of a biallelic simultaneous transcription is rare^27^.

### Fraction of expressing cells causes gene dosage imbalance

**Fig. 4:**
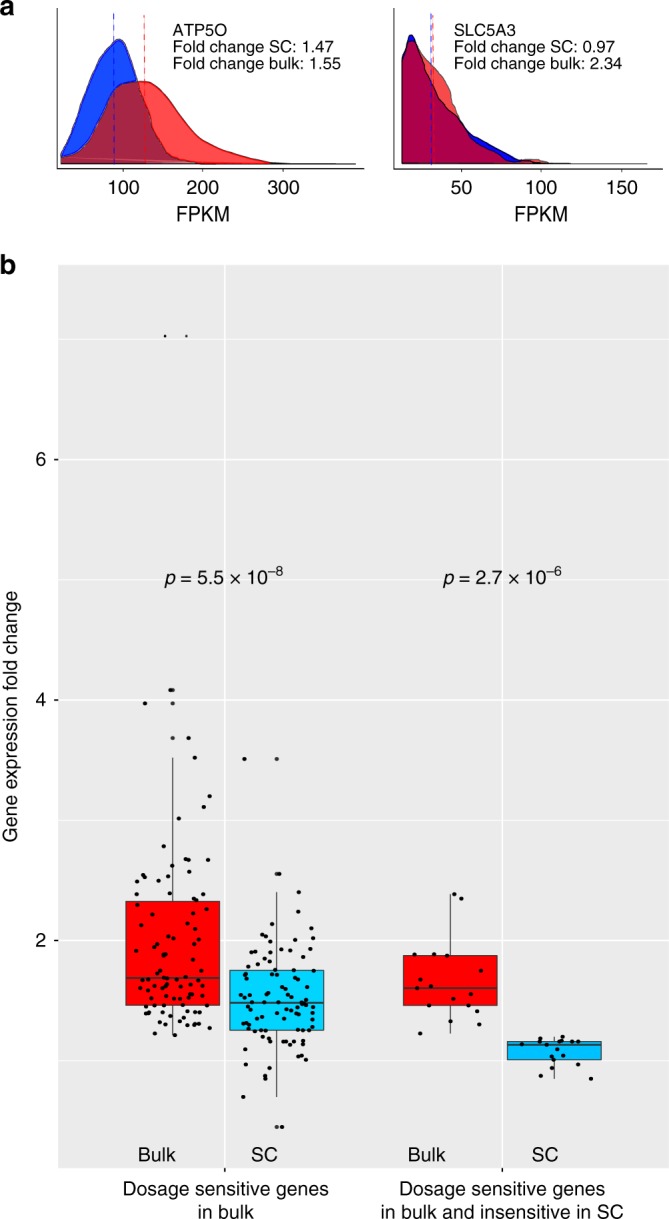
Fold change expression comparison in bulk and single-cell study. **a** Left: Distribution of expression levels of *ATP5O* in diploid (blue) and T21 (red) single cells. The gene presents with the typical trisomy gene dosage effect meanT21/mean = 1.5 as observed in the bulk (FCbulk = 1.5). Right: Distribution of expression levels of *SLC5A3* in diploid and T21 single cells. The two distributions are similar and the gene does not present the typical gene dosage effect as observed in the bulk (meanT21/meanD = 1, FC_bulk_ = 2). **b** Left: Comparison of expression fold change for dosage sensitive genes in the bulk (FC_bulk_ > 1.2, 94 genes) and SC of twins discordant for T21. Right: Comparison of expression fold change in bulk and SC for a subset of bulk-dosage sensitive genes presenting with a non-dosage sensitive effect in SCs (insensitive in SC) (0.8 < SC FC < 1.2, 17 genes). Boxplot: horizontal lines indicate medians; upper and lower boxes indicate first (25th percentile) and third quartiles (75th percentile); whiskers indicate first quartile—1.5 IQR (interquantile range = first–third quartile) and third quartile + 1.5 IQR. Source data are provided as Source Data file

In bulk studies, triplicated genes show overall the expected 1.5 gene expression fold change (trisomic vs diploid, FC). This observation has been attributed to the increased amount of transcripts produced by the triplicated genes. Our study allows a more detailed interpretation of gene dosage imbalance in aneuploidies. As an example, considering the previous bulk study on fibroblasts from the discordant twins for T21^30^, FC of *SLC5A3* between normal and trisomic state is different between the bulk sample (FC = 2.34) and across the single cells (mean(FC) = 0.97) conversely, *ATP5O* has a FC of 1.55 in bulk and 1.47 in single cells (Fig. 4a). In general, we observed that dosage sensitive genes in the bulk have a significantly lower FC expression in single cells. FC for 94 chr21 dosage sensitive genes in the bulk sample is superior to 1.2 (T21/N) whereas many genes have a reduced or no gene dosage effect at the single-cell level (Fig. 4b). The explanation can be provided considering the stochastic nature of gene expression. From the master equation of a 2-state promoter, the solution for genes transcribed in non-overlapping bursts (i.e. rate of gene inactivation » decay rate) takes the form of a negative binomial^31^. Considering the observed expression ratio k/2 (= 3/2 in triplicated genes versus diploid), we derived the hyperbolic equation (see Supplementary Note S1):

1 $$\frac{{E({\mathbf{s}}_k)}}{{E({\mathbf{s}}_2)}} = \frac{{R_k\bar s_k}}{{R_2\bar s_2}} = \frac{k}{2}$$

where **s** is the distribution of expression levels of g in a bulk of cells, *R* is the fraction of cells expressing g and $\bar S$ is the average (zero truncated) expression of g. Equation (1) reveals the inverse proportionality between the mean FC in gene expression $\frac{{\bar s_k}}{{\bar s_2}}$ and the K-somic/Diploid ratio of the number of expressing cells $\frac{{R_k}}{{R_2}}$.

The mathematical model shows that genes in three copies with low expression level in single cells tend to be expressed in more trisomic cells than diploid cells. This theoretical result, derived from the 2-state burst-like model of transcription applied to 3 alleles, supports the general hypothesis that a component of bulk gene dosage imbalance of copy altered genes is generated by the increased number of cells expressing these genes at a given time point.

**Fig. 5:**
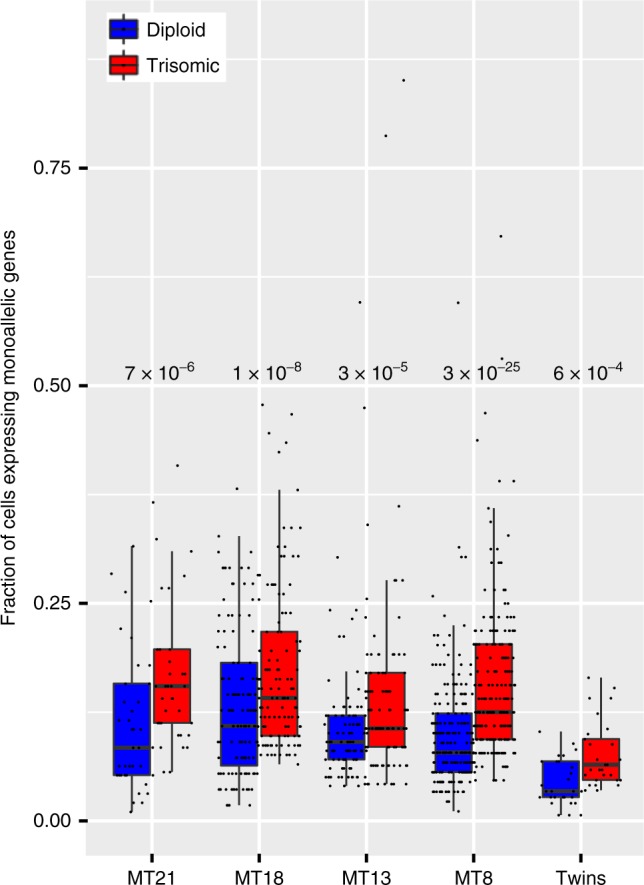
Higher fraction of expressed cells for supernumerary chromosome genes. In all trisomies an increased fraction of single trisomic cells expressing supernumerary monoallelic genes is detectable. *P*-values in the figure indicate the respective statistical significance of the comparisons between diploid and trisomic cells. Blue—fraction of diploid single cells. Red—fraction of trisomic single cells. Boxplot: horizontal lines indicate medians; upper and lower boxes indicate first (25th percentile) and third quartiles (75th percentile); whiskers indicate first quartile—1.5 IQR (interquantile range = first–third quartile) and third quartile + 1.5 IQR. MT = Mosaic Trisomy.  Source data are provided as Source Data file

Along this hypothesis, we estimated the fraction of trisomic cells expressing each triplicated gene *R*_3_, and compared to the fraction of cells of the corresponding diploid sample *R*_2_. We consider a gene as expressed if: (i) the total number of cells expressing the site within the gene is ≥10, (ii) each cellular ASE observation has an RPSM score ≥ 20 (Reads Per Site Per Million^17^). The genes were classified according to their prevalence of monoallelic expression (monoallelic, intermediate, biallelic) as previously defined. Biallelic or intermediate genes did not show statistically significant differences in the ratio of the fractions of expressing cells $\frac{{R_3}}{{R_2}}$. In contrast, Twins and mosaic T21, T18, T8, T13 showed a statistically significant higher fraction of expressing trisomic than diploid cells of monoallelic genes on the supernumerary chromosomes with respective p-values 6 × 10^−4^, 7 × 10^−6^, 1 × 10^−8^, 3 × 10^−25^, 3 × 10^−5^ (paired Wilcoxon signed-rank test, two-sided, Fig. 5).

**Fig. 6:**
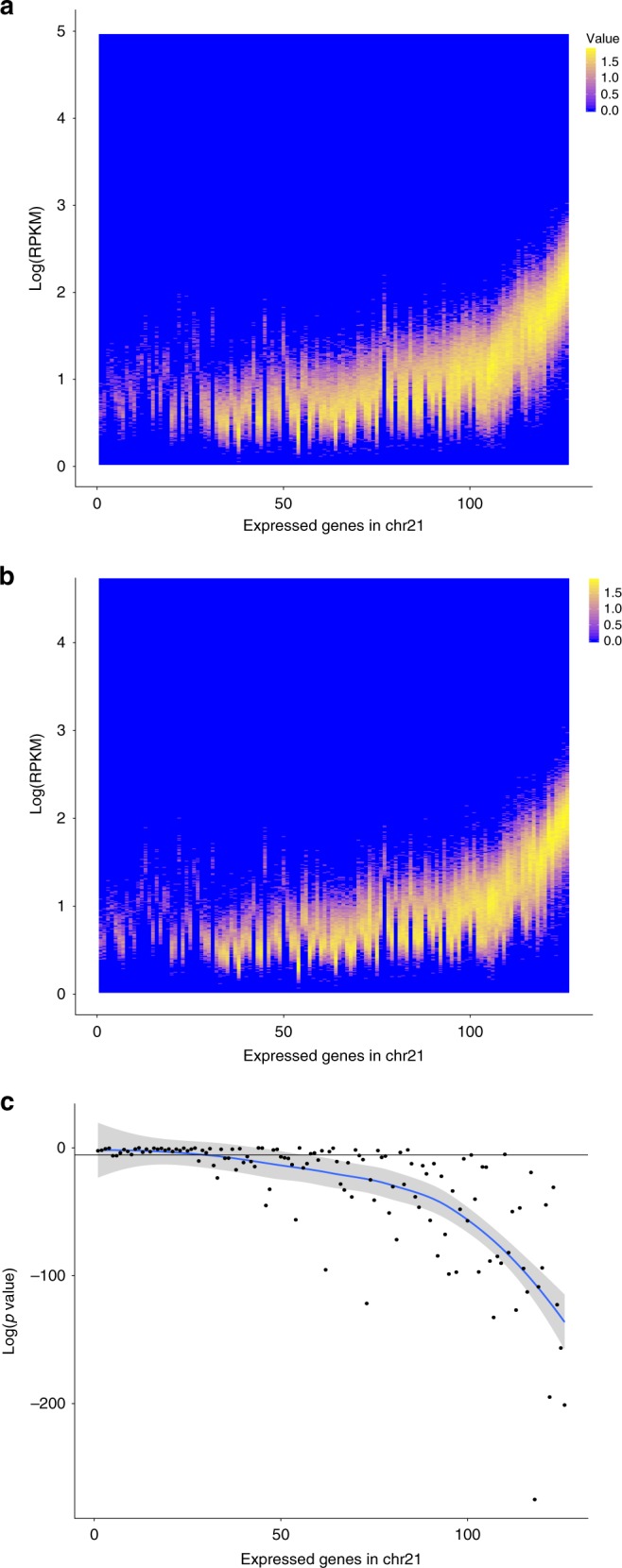
Distribution of expression levels of chr21 genes in trisomic and normal cells. **a**, **b** Distribution of expression levels (log(RPKM) on the *y*-axis, counts are color coded) of genes in chr21 (on the *x*-axis, sorted by increasing average expression) in 4939 trisomic and 3801 diploid cells, respectively. **c** Significance of the gene-matched (on the x-axis) statistical comparison (on the *y*-axis) of trisomic (**a**) and diploid (**b**) distributions. Low expressed genes (on the left) tend to have a similar distribution in trisomic and diploid cells (no significant difference) as opposed to the average and highly expressed genes (on the right). Source data are provided in the public repository

**Fig. 7:**
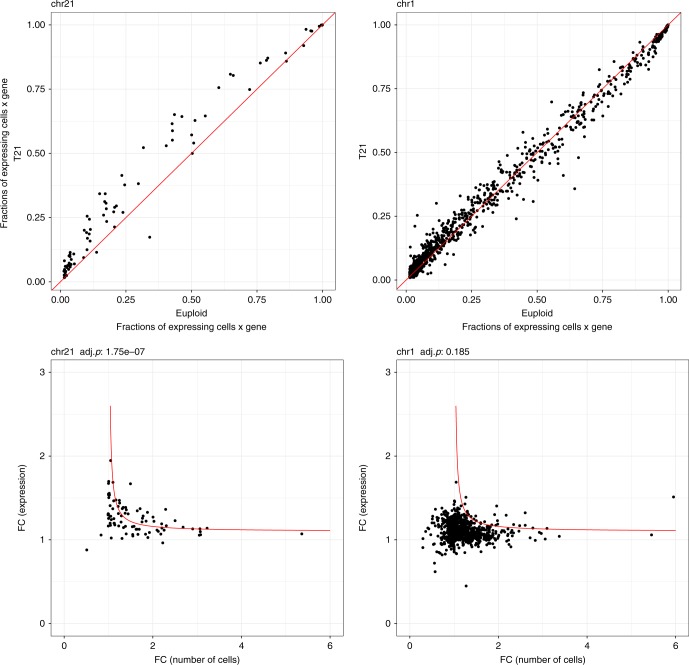
Higher fraction of expressing cells of trisomic genes in 8740 single fibroblasts. Upper row, left: (*y*-axis) distribution of fraction of trisomic cells expressing chr21 genes; (*x*-axis) distribution of fraction of diploid single cells expressing chr21 genes; right: data for chr1 as control. Lower row, left: T/D ratio of number of expressing cells and T/D ratio of single-cell expression of genes in chr21 are inversely correlated (Spearman correlation); right: data for chr1 as control. Cells with >5 reads and genes expressed in >50 cells have been considered. Red line is to guide the eye (see text for details). Source data are provided in the public repository

**Fig. 8:**
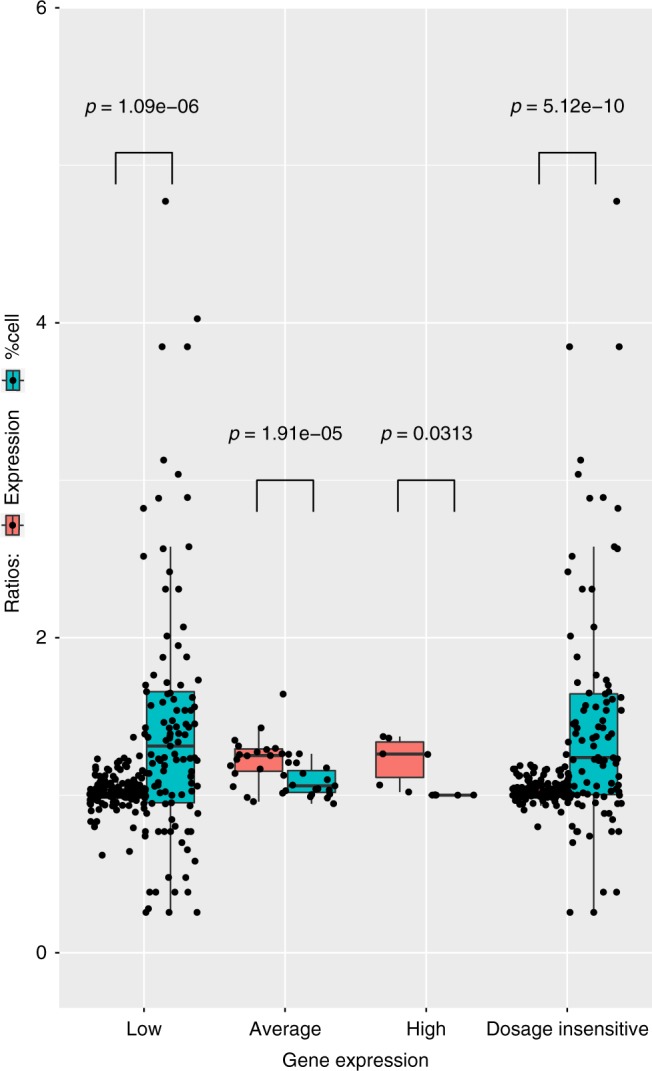
Components of gene dosage imbalance in trisomy 21 using 8740 single fibroblasts. Left: Gene dosage imbalance components in low (<3 FPKM), medium (3 FPKM< and <15 FPKM) and high (>15 FPKM) expressed genes. For low expressed genes, dosage imbalance is mainly driven by the increased fraction of trisomic cells expressing these genes compared to diploid. For medium and highly expressed genes, the dosage imbalance is mainly driven by the trisomic/diploid FC expression per cell while no significant difference in the fraction of cells can be detected. Right: statistically significant differences of trisomic/diploid ratio of fraction of expressing cells in non-dosage sensitive genes in single cells (0.8 < FC < 1.2). Boxplot: horizontal lines indicate medians; upper and lower boxes indicate first (25th percentile) and third quartiles (75th percentile); whiskers indicate first quartile—1.5 IQR (interquantile range = first–third quartile) and third quartile + 1.5 IQR. Source data are provided in the public repository

We validated these results with an additional and independent experiment using the chromium single-cell controller (10X Genomics)^32^, a droplet-based system for scRNAseq. We processed 3801 diploid single cells and 4939 T21 single cells derived from the monozygotic twins fibroblasts discordant for T21. After random selection of an equal number of trisomic and diploid cells (3800) and normalization with respect to the total number of UMIs per cell, for each chr21 gene we calculated the expression distribution in normal and trisomic cell, respectively (Fig. 6a, b). We compared the gene-matched distributions and, in agreement with Eq. (1), we noticed that low expressed genes do not statistically differ in term of expression levels between trisomic and normal cells (Mann-Whitney test). Conversely average and highly expressed genes present with a significantly different distribution (Fig. 6c). Additionally we confirmed that, for chr21 genes only, FC expression of genes and respective FC of number of expressing cells fit the hyperbolic model of Equation (4) (*p* = 1 × 10^−7^, Spearman correlation, Fig. 7 and Supplementary Fig. 8). As expected, for all genes in the autosomal chromosomes but the trisomic ones, the fraction of expressing cells was equivalent in both trisomic and diploid cells (Supplementary Fig. 8). To combine the two dimensions together, we calculated single-cell expression distribution and the fraction of expressing cells for each expressed chr21 gene in the trisomic and normal cells of the discordant twins. Again in agreement with Eq. (1) and the results reported in Fig. 6, we observed that the distribution of low transcribed genes was not significantly different in trisomic versus normal as it was for average and high transcribed genes (Fig. 8). More specifically, we observed that gene dosage insensitive genes (0.8 < FC < 1.2), tend to exhibit a higher median fraction of trisomic vs diploid expressing cell ratio (1.3, *p* = 5 × 10^−10^, Mann–Whitney U test) (Fig. 8). This result indicates that the fraction of expressing cells is the main component of gene dosage imbalance for such genes. Notably, low expressed genes in chr21 (183 genes) showed a higher $\frac{{R_3}}{{R_2}}$ than intermediate (22 genes) and highly expressed genes (9 genes) (Fig. 8). We conclude that for low expressed genes, the gene dosage imbalance is mainly driven by the higher fraction of T21 expressing cells (*p* = 1 × 10^−6^, Mann–Whitney U test, Fig. 8). Conversely, for intermediate and highly expressed genes, the main component of gene dosage effect is the higher expression of triplicated genes in each single cell (*p* = 2 × 10^−5^ and *p* = 0.03 Mann–Whitney U test, respectively, Fig. 8).

## Discussion

Gene dosage imbalance caused by CNA is generally interpreted as the altered production of transcripts per cell. The typical example is represented by trisomies where several studies on mouse and human samples reported the classical 1.5 average gene expression fold change^7,30,33–39^. The investigation of this phenomenon at single-cell level opened a more complex scenario. As a first observation, we detected reduced or no dosage effect at all for some genes on a single-cell level, compared to the expected fold change from our previous bulk study in T21. Moreover, ASE exploration of the supernumerary chromosome genes in our isogenic models of trisomies showed clear random monoallelic patterns of expression as already observed in diploid cells^17,26,28^. We confirmed that these patterns follow a random allelic selection model by observing that the number of observations expressing the duplicated allele was indeed twice the number of those expressing the single allele. As previously shown^17,26^, we observed that the monoallelic prevalence of expression (the fraction of cells in which the gene appears as expressed by one allele only) is negatively correlated with the level of expression of the respective genes. Finally, we observed that the increased fraction of trisomic cells vs. diploid presenting with active expression of supernumerary chromosome genes is contributing to the average dosage imbalance of all the trisomies analyzed in this study. This effect is more evident for low expressed/monoallelic genes.

The theoretical application of the 2-state burst-like transcriptional model to trisomies provides an explanation of these observations (Eq. (1)). For average and highly expressed genes (i.e., housekeeping and maintenance of cell function), transcriptional bursting events are frequent in both diploid and trisomic single cells. For these genes, the model predicts an increment of RNA molecules at single-cell level and in all single cells. Accordingly, we observed no significant difference in the fraction of expressing cells. Conversely, for low expressed genes with a low bursting frequency and burst size, the model predicts that the presence of additional alleles increases the number of random transcriptional events in more single cells. Being rare events hardly happening simultaneously in the same cells, the transcriptional bursts of low transcribed duplicated genes do not lead to a detectable increased amount of RNA quantity in the single cell but instead to an increased monoallelic expression prevalence in a higher fraction of expressing cells with respect to diploid controls. We have shown that this model fits quite closely the experimental data describing a whole spectrum between the two above-mentioned extremes (Fig. 7). Duplicated genes with average expression will present gene dosage imbalance with a combination of RNA accumulation and fraction of expressing cells accordingly to the inverse relationship described by Eq. (1).

This observation may have a significant impact on the understanding the molecular pathophysiology of aneuploidies. As an example, transcription factors (TF) have in general a lower expression level than non transcription factor genes^40^. TF dosage imbalance leading to an increased number of a activated cells could be crucial in the following (the list is not exhaustive): (1) different fractions of cells producing increased level of subunits of multimeric proteins may result in abnormal stoichiometry^41,42^; (2) abnormal number of cells with cell surface receptors and ligands that may results in a disturbed developmental fate^43,44^; (3) abnormal number of transporter molecules in the tissue resulting in metabolic disturbances^45^; (4) excess of cell adhesion molecules that may increase cellular adhesiveness and differential fate of a tissue^46^; (5) alteration in the production, concentration and diffusion of morphogens in the tissue and consequent abnormal cellular proliferation and development of aberrant cellular and tissue structures^47^. Furthermore, the unbalanced expression of low expressed copy altered regulatory long non-coding RNAs and microRNAs in a fraction of cells may also contribute to the disturbance of the regulatory repertoire of other cells, particularly during embryogenesis^48^. Indeed many of these phenotypes may manifest during the early embryonic development stages where a precise and delicate balance among gene pathways dedicated to coordinate cell-to-cell interactions as well a as specific partition among cell types must be maintained^49^. Additionally this effect can be mediated by the duplication of regulatory regions that modulate gene expression through specific regulatory variants. eQTLs in trisomic regions have 4 possible states (AAA, AAB, ABB, BBB) instead of the canonical (AA, AB, BB) in the diploid genome. This additional degree of freedom might modulate the spectrum of gene dosage imbalance in term of RNA accumulation and fraction of expressing cells and contribute to the considerable phenotypic variability among affected individuals. More generally, we propose that the spectrum of gene dosage may contribute to phenotypes related to Copy Number Alteration, including Copy Number Variants (CNVs) and somatic partial aneuploidies typical of cancer cells^50^. Based on this considerations, it is straightforward to hypothesize that strong regulatory variants on low expressed genes may induce gene dosage diversity in general population. Time-series single-cell RNAseq studies in normal and aneuploid differentiating embryos are needed to reveal how the spectrum of gene dosage imbalance determines individual phenotypic features.

## Methods

### Ethical statement

The study was approved by the ethics committee of the University Hospitals of Geneva, and written informed consent was obtained from both parents of the twins.

### Samples

We used six different cell lines of skin fibroblasts from six individuals: two samples are from a pair of monozygotic twins discordant for T21^25^; four were cell lines obtained from Coriell and derived from individuals mosaics for T21: CM05287, T13: GM00503, T18: AG13074, T8: GM02596 (https://www.coriell.org/). DNA samples from peripheral blood were obtained from the parents of the monozygotic twins. Cell lines from mosaic individuals T8, mosaic T13, mosaic T18, were purchased from Corriel, and sample from mosaic T21 was kindly provided by Prof. Dean Nizetic. We captured in total 928 single-cell fibroblasts (484 diploid and 444 Trisomic) using the Fluidigm C1 technology. In addition we employed an alternative single-cell RNA-seq protocol based on 10X Genomics technology (Chromium Single cell 3’ Solution protocol^32^) to capture 8740 single cells (3801 diploid and 4939 trisomic single cells) (Supplementary Table 1).

### Analysis of genome-matched samples

The comparison of transcriptional profiles of unrelated individuals is complicated by the substantial genetic variability^30^. Notably, in this study, we sought to eliminate the inter-individual bias by comparing diploid and trisomic single-cell fibroblasts from individuals with mosaicism for the relevant trisomies (T8, T13, T18, T21) and by using single-cell fibroblasts from monozygotic twins discordant for DS (T21) (Supplementary Fig. 1 and Supplementary Table 1).

### Cell culture

Cells were cultured in DMEM GlutaMAX™ (Life Technologies) supplemented with 10% fetal bovine serum (Life Technologies) and 1% penicillin/streptomycin/fungizone mix (Amimed, BioConcept) at 37 °C in a 5% CO2 atmosphere. The day before the single-cell capture experiment; cells were trypsinized (Trypsin 0.05%-EDTA, Life Technologies) and replated at a density of 0.3 × 10^6^ cells/100-mm dish.

### Fluorescence in situ Hybridization

Fluorescent in situ Hybridization (FISH) analysis was performed on cultured-interphase nuclei with 2 set of probes: two locus specific probes on chromosome 13 (Vysis RB_1;13q14_ locus) and chromosome 21 (Vysis, D_21_S_342_/D_21_S_341_/D_21_S_259_ contig probes), and two alpha satellite centromere probes for chromosome 8 (Vysis, D8Z1) and chromosome 18 (Vysis, D18Z1). The experiments were carried out according to manufacturer’s instructions (Aneuvysion, VYSIS Inc.). For each sample 150 interphase nuclei were examined to evaluate the mosaic rate.

### Whole-genome sequencing

Genomic DNA was extracted for five individuals using a QIAamp DNA Blood Mini Kit (Qiagen) and fragmented by Covaris to peak sizes of 300–400 bp. Libraries were prepared with TruSeq DNA kit (Illumina) using 1 µg of gDNA and sequenced on an Illumina HiSeq 2000 machine with 2 × 100-bp^17^. All experiments were performed using the manufacturer’s protocols. All samples provided with an whole genome average coverage around 25×. For each individual, raw whole genome DNA sequences were analyzed using an in-house pipeline. Briefly, we used the BurrowsWheeler Aligner (BWA mem v.0.7.10) to align the sequencing reads (fastq) to the human reference genome (GRCh37/hg19). We used SAMtools v.1.4^51^ to remove paired-end duplicates and pile up the remaining reads. BCFtools v.1.4 was used to call the SNVs and Annovar (2016Feb01)^52^ for the annotation. SNVs with quality score <100 where excluded from the analysis. All putatively biased sites with low mappability (i.e. in repeated or bad quality regions) were removed from the analysis as suggested by Panousis et al.^53^. Similarly to^18,19^, we only used uniquely mapped reads for SNV calling.

### Single-cell capture (C1 Fluidigm)

Single-cell capture was performed by C1 single-cell auto prep system (Fluidigm) following the manufacturer’s instructions^17^. The microfluidics circuit used was the C1™ Single-Cell mRNA-seq IFC, 17–25 µm. All 96 chambers were inspected under an inverted phase contrast microscope; only chambers containing a non-damaged single cell were considered for downstream analysis. For the cell lysis and cDNA synthesis, we used the SMARTer Ultra Low RNA kit for Illumina Sequencing (version 2, Clontech) and a C1 Auto Prep System instrument (Fluidigm) with the original mRNA Seq Prep script provided by the manufacturer (1772×/1773×, Fluidigm). We assessed cDNA quality on 2100 Bioanalyzer (Agilent) with the high sensitivity DNA chips (Agilent) and quantified the cDNA using Qubit dsDNA BR assay kit (Invitrogen). Sequencing libraries were prepared with 0.3 ng of pre-amplified cDNA using Nextera XT DNA kit (Illumina) according to manufacturer’s instructions. Libraries were sequenced on an Illumina HiSeq2000 machine as 100 bp reads single-end.

### GemCode single-cell libraries preparation and sequencing

We captured in total 3801 diploid and 4939 trisomic single-cell fibroblasts from the monozygotic twin pair using the Chromium System powered by GemCode Technology (10x Genomics). Single-cell RNA-seq libraries were generated using the Chromium Single Cell 3' Reagent Kit version 2 (10x Genomics) according to the manufacturer’s instructions. Briefly, the concentration of trypsin dissociated fibroblasts was set to 1500 cells/µl of culture medium (Dulbecco’s Modified Eagle Medium (DMEM), 10% FBS) and 5000 individualized cells were flown per channel following the recommendation of the manufacturer. All libraries were quantified by Qubit (Invitrogen) and by quantitative real-time PCR using the PCR-based KAPA Library Quantification Kits for Illumina platforms (Kapabiosystems). Size profiles of the pre-amplified cDNA and sequencing libraries were assessed using a 2100 BioAnalyzer (Agilent) with a High Sensitivity DNA chip kit (Agilent). Barcoded libraries were sequenced with an HiSeq 4000 (Illumina) as paired-end 100 bp reads as recommended by 10x Genomics. The proprietary software CellRanger (10x Genomics) with default parameters was used in order to demultiplex the samples and quantify the abundance of mRNA molecules (UMI - Unique Molecular Identifier). Processed data were analyzed using custom R scripts.

### C1 Single-cell RNA-sequencing

For single cells capture with the Fluidigm C1 microfluidics system, SMARTer Ultra Low RNA kit for Illumina sequencing (version 2, Clontech) was used for cell lysis and cDNA synthesis. 0.3 ng of pre-amplified cDNA, was used for the library preparation with the Nextera XT DNA kit (Illumina) as described^17^. Libraries were sequenced on an Illumina HiSeq2000 sequencer as 100 bp single-ended reads. RNA sequences were mapped with GEM^54^. Uniquely mapping reads were extracted by filtering for mapping quality ≥ (MQ ≥ 150). For FPKM expression quantification an in-house algorithm was used with GENCODE v19 as reference. Cells with less than 1o million reads and/or cells with <10% of expressed genes (total number of 56680 genes) were excluded from the analyses. For each individual, ASE of each heterozygous SNP identified by WGS has been calculated using in-house developed Python scripts. Data have been analyzed using custom R scripts.

### Allele-specific expression

Cellular Allelic Specific Expression (ASE) of each heterozygous site was calculated in the diploid and trisomic fraction of the genome of each single cell per individual using two different formulas.

Diploid fraction:

$${\mathrm{ASE}}_i = \frac {{n}{{\mathrm{reads}}({\mathrm{REF}},i)}}{{{n}{\mathrm{reads}}({\mathrm{REF}},i) + {n}{\mathrm{reads}}({\mathrm{ALT}},i)}}$$

Trisomic fraction:

$${\mathrm{ASE}}_i = \frac{{{n}{\mathrm{reads}}({\mathrm{DA}},i)}}{{{n}{\mathrm{reads}}({\mathrm{SA}},i) + {n}{\mathrm{reads}}({\mathrm{DA}},i)}}$$

where *n*reads is an operator giving the number of reads covering the site i, mapped according to the REFerence or the ALTernative allele (euploid) or to the Double or Single Allele (trisomic).

In both cases, ASE values range from 0 to 1 (Supplementary Fig. 5). We consider 0 ≤ ASE ≤ 0.1 as the signature of monoallelic expression of the Alternative allele (euploid) or Single allele (trisomic). Conversely 0.9 ≤ ASE ≤ 1 indicates monoallelic expression of the Reference allele in the case of diploid cells or of the Double allele in the case of (trisomic cells). ASE from 0.1 to 0.9 is an indicator of biallelic expression.

### Single cells identification in mosaic populations

We developed a computational procedure to distinguish diploid from trisomic single cells in mosaic populations. Using an iterated k-means (k = 2) approach, we combined ASE profiling and expression data of every expressed site in the supernumerary chromosomes to classify each single cell as diploid or trisomic. At the beginning the algorithm applies a k(=2)-means clustering to obtain the first partition of normal and trisomic cells using as features average minmax normalized gene expression x cell and average ASE x cell. The rationale is that average gene expression of genes in the supernumerary chromosome is on average higher than the corresponding gene in the diploid cell with the same genetic background (i.e., no difference in regulatory regions). Moreover, as previously shown, average ASE calculated with respect to the double allele in trisomic cells (see Methods) is higher than the corresponding ASE in diploid cells. However, since we do not know a priori the allele in two copies (double) the accuracy of the first partition is expected to be quite poor. Therefore the next step is to estimate for each heterozygous site the double allele using ASE imbalance of single cells. More specifically, the average ASE x site is calculated across all single cells tagged as trisomic in the previous iteration and the double allele assigned to each site as the max expressed allele. Once this estimation is done, the improved ASE prediction is used in a new k(=2)-means iteration to improve the partition of trisomic and diploid cells (the second feature is again the average gene expression). K-means cell clustering and double allele estimation are repeated until convergence is reached (i.e. trisomic and diploid cell clusters are stable, no reassignment) (Supplementary Fig. 4).

### Fluidigm C1 multiple cells (doublets) detection

In our Fluidigm C1 based protocol, we set two checkpoints where double cells (doublets) are identified and eliminated. First, during the capturing procedure, doublets are identified by visual inspection under the microscope, and eliminated from further analysis. Second, after RNA sequencing and ASE analysis, potential double cells of female individuals are eliminated based on the study of X chromosome haplotype expression. For each cell, the expressed haplotype is estimated by calculating the allelic ratio of each heterozygous site in the X chromosome as identified by whole genome sequencing. Sites in the pseudoautosomal regions (PAR1 chrX:60001–2699520, PAR2 chrX:154931044–155260560) and known escapee genes are a priori excluded. The estimated haplotype of each cell was compared to all the others through correlation based hierarchical clustering. Cells expressing concordant and discordant haplotypes results in a correlation near 1 and −1 respectively. Doublets simultaneously expressing both discordant haplotypes cluster around the absolute correlation of 0.5 and are excluded from further analysis (Supplementary Fig. 3).

### Allele dropout control

To reduce the potential bias induced by allele dropout, we have utilized the RPSM metric (Reads per site per million mapped reads)^17^. Through split cell RNA experiments based on ERCC RNA spike-in mix (Ambion)^17^, we identified the threshold RPSM = 20 to drastically reduce false positive monoallelic ASE calls (Supplementary Fig. 2). Additionally we only consider heterozygous SNV sites covered by at least 16 reads to further minimize possible allele dropout effects^55^.

### Reporting summary

Further information on research design is available in the Nature Research Reporting Summary linked to this article.

## Supplementary information

Supplementary Information

Reporting Summary

Source Data

## Data Availability

Fluidigm C1 Sequencing data for Discordant T21 Twins and mosaic T18 and T8 are available in the Gene Expression Omnibus (GEO) data repository (accession no. GSE123028). Sequencing data for Discordant T21 (10X Genomics), Mosaic T21 and T13 (Fluidigm C1) are available in the Gene Expression Omnibus (GEO) data repository (accession no. GSE135500). All other relevant data are available upon request.

## Code availability

Python and R code used in this study is available upon request.
