# Supplementary material for: Single cell transcriptome in aneuploidies reveals mechanisms of gene dosage imbalance
Source: Nat Commun. 2019 Oct 3;10:4495. doi: 10.1038/s41467-019-12273-8 (PMC6776538; doi:10.1038/s41467-019-12273-8)
Supplement: Supplementary file 1 — Supplementary Information [file 41467_2019_12273_MOESM1_ESM.pdf]

Single cell transcriptome in aneuploidies reveals mechanisms of gene dosage imbalance

Georgios Stamoulis[1], Marco Garieri[1], Periklis Makrythanasis[2], Audrey Letourneau[1], Michel Guipponi[2], Nikolaos Panousis[1], Frédérique Sloan-Béna[2], Emilie Falconnet[1], Pascale Ribaux[1], Christelle Borel[1], Federico Santoni[4,§,*], Stylianos E Antonarakis[1,2,3,§,*]

[1]Department of Genetic Medicine and Development, University of Geneva Medical School, 1211 Geneva 4, Switzerland, [2]Geneva University Hospitals, Service of Genetic Medicine, 1211 Geneva 4, Switzerland, [3]iGE3 Institute of Genetics and Genomics of Geneva, University of Geneva, 1211 Geneva 4, Switzerland, [4]Service of Endocrinology, Diabetes and Metabolism, University Hospital of Lausanne - CHUV, Lausanne 1011, Switzerland.

[§]These authors contributed equally
[*]Corresponding Authors

SUPPLEMENTARY FIGURE 1 2
SUPPLEMENTARY FIGURE 2 4
SUPPLEMENTARY FIGURE 3 5
SUPPLEMENTARY FIGURE 4 6
SUPPLEMENTARY FIGURE 5 8
SUPPLEMENTARY FIGURE 6 9
SUPPLEMENTARY FIGURE 7 10
SUPPLEMENTARY FIGURE 8 11
SUPPLEMENTARY TABLE 1 13
SUPPLEMENTARY NOTE 1 14

Supplementary Figure 1

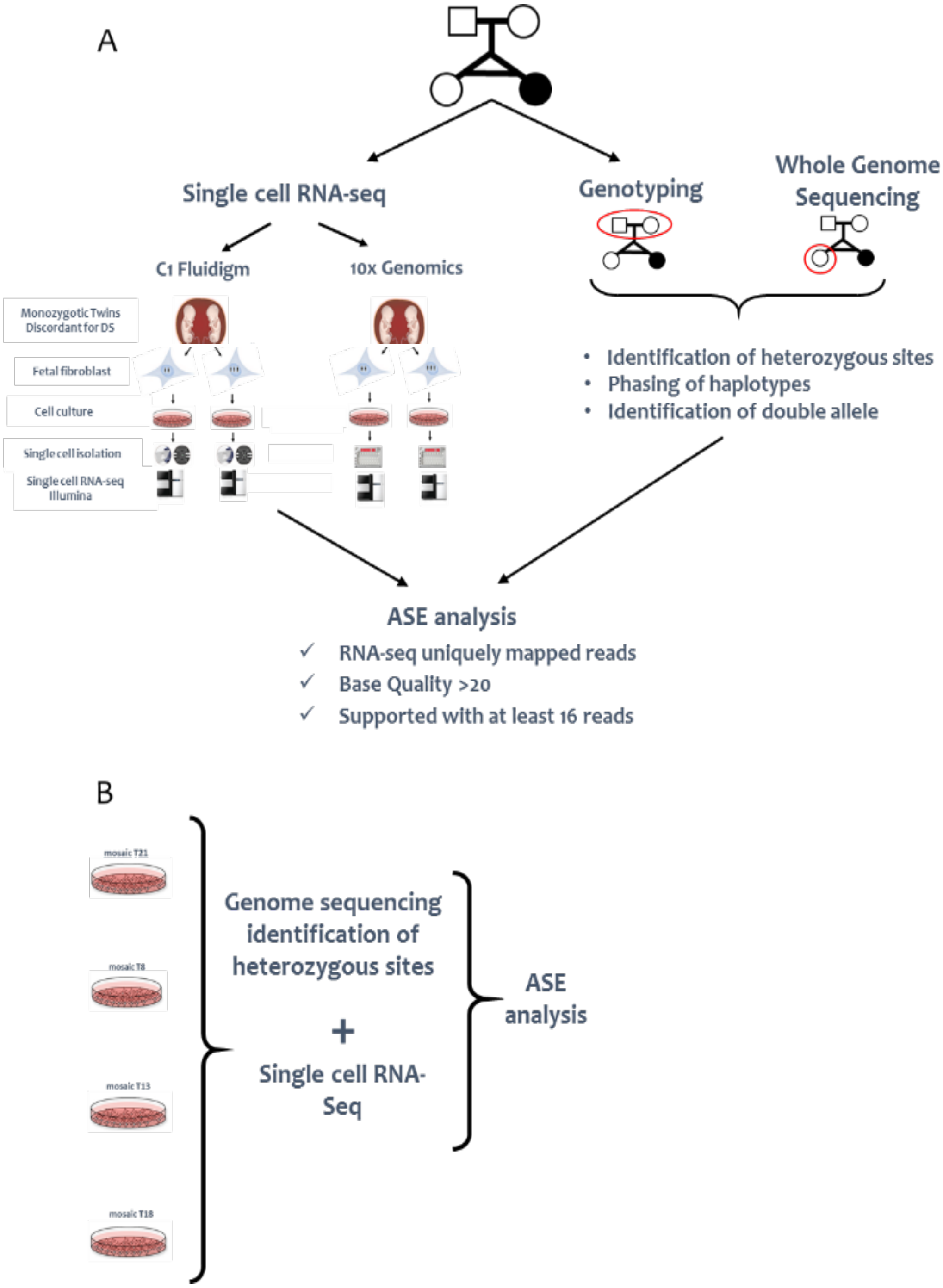

Supplementary Figure 1. Flowchart and Strategy of the study: A) Strategy for ASE investigation of monozygotic twins discordant for T21. Single cell RNA-seq was performed on fibroblasts derived from monozygotic twins discordant for DS with two different single cell RNA-seq methods (C1 Fluidigm, 10x Genomics). All heterozygous sites were identified through WGS of the euploid twin. Genotyping information of the parents was used to phase the haplotypes and thereby identify the double allele for each heterozygous site. ASE analysis was performed combining single cell RNA-seq and WGS. B) Strategy for ASE investigation of mosaic trisomic cells. WGS of all available trisomies was performed for the identification of heterozygous sites. In parallel single cell RNA-seq for each trisomy was performed. ASE analysis of all trisomies was done combining single cell RNA-seq and WGS.

Supplementary Figure 2

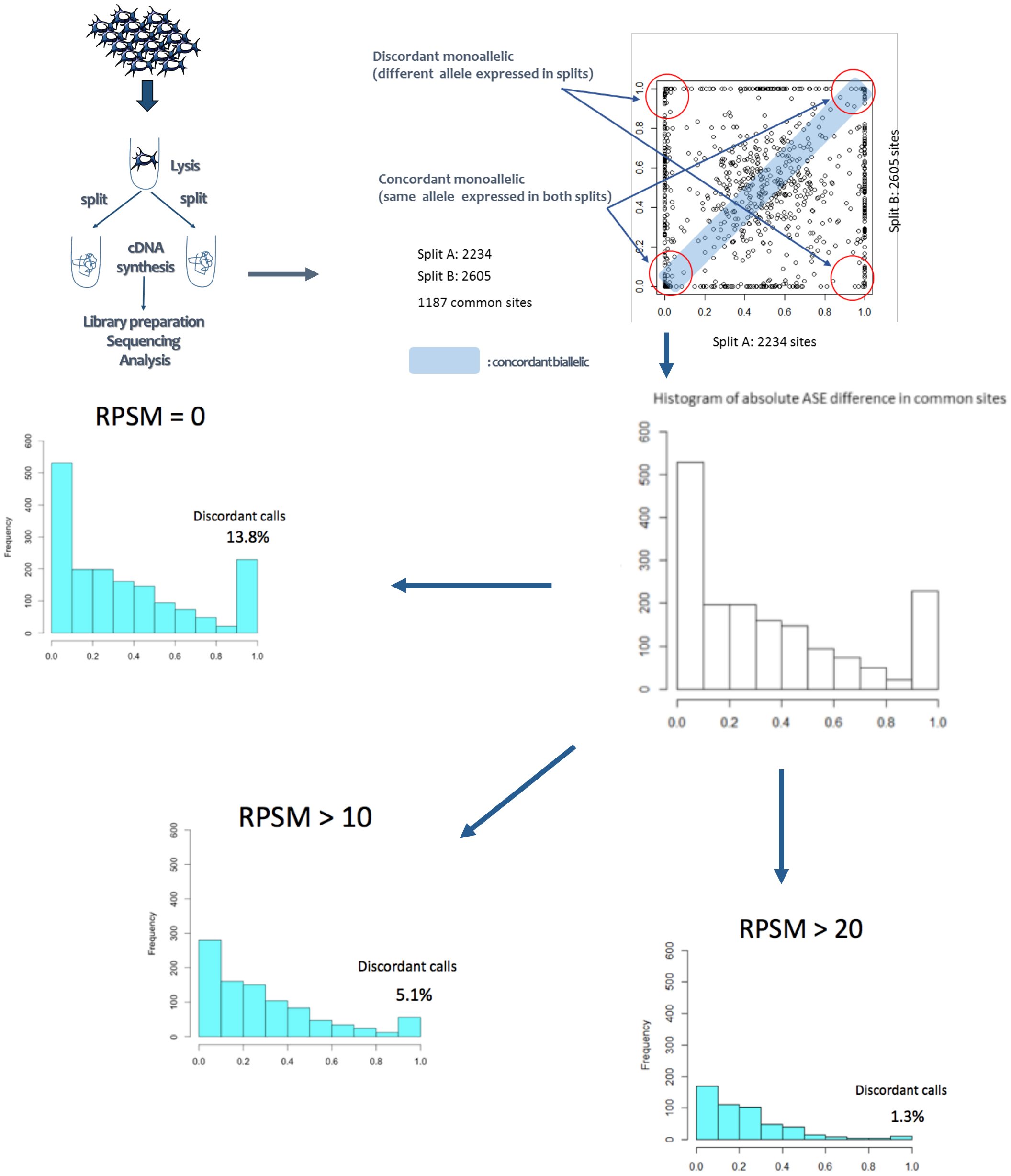

Supplementary Figure 2: **Split cell experiment for the establishment of RPSM threshold.** First we manually split the content of a single cell and performed cDNA synthesis in separate tubes. Independently, we afterwards prepared libraries for the two splits, and sequenced both of them. After ASE estimation, we focused on the common sites detected in the split cells. Common sites discordant for ASE are a bias introduced by the allele drop-out effect. By calculating the absolute distribution of ASE differences between common sites we observed that discordant monoallelic sites driven by allelic dropout almost vanished (<1.5%) at RPSM=20. We replicated this result in additional 12 single cells.

Supplementary Figure 3

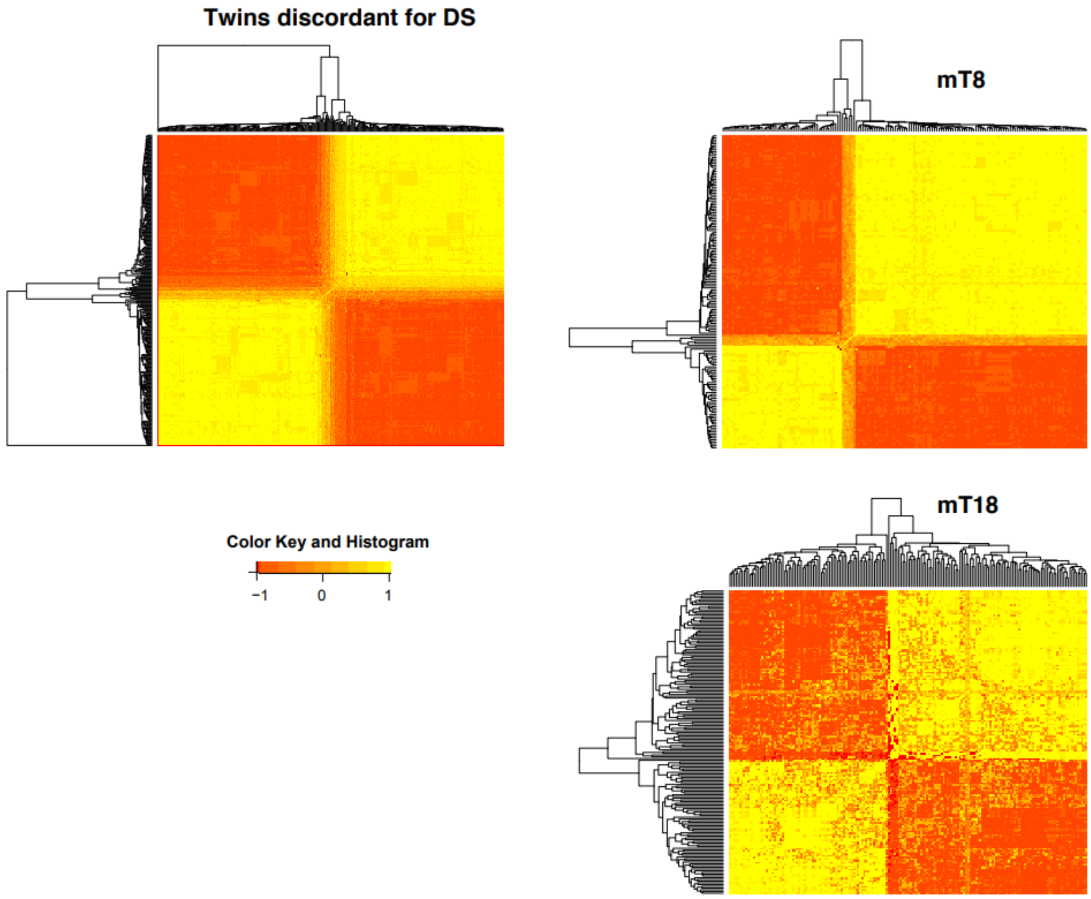

Supplementary Figure 3. Heat map of unsupervised hierarchical clustering using cell-to-cell pairwise Pearson correlation coefficients of the allelic ratios as calculated in single cells from 4 female individuals (mosT18, mosT8, and monozygotic twins discordant for T21), in order to identify possible double cells (doublets). The heat map separated the cells expressing one haplotype from cells expressing two haplotypes (cluster of cells with correlation close to zero are labeled as doublets). Pearson correlations range from -1 (red) to 1 (yellow).

Supplementary Figure 4

A

Step 1

cells are clustered based on FPKM only
(Allelic Ratio is random)

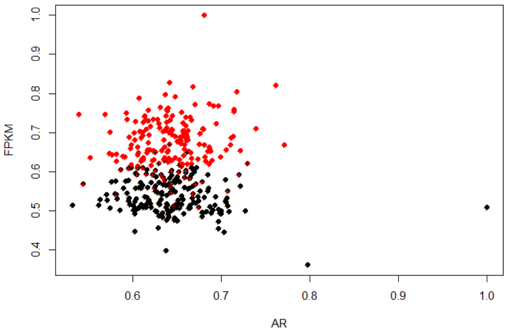

Step 2

Putative T21 cells are used to evaluate the “Double Allele” and the Allelic Ratio (Double/Total)

		Cell 1	Cell 2	Cell 3	...	Total	Double
Chr1:19239003	Ref	34	56	48	...	1345	**Ref**
	Alt	10	23	19	...	598	
Chr1:19254067	Ref	14	17	9	...	139	**Alt**
	Alt	50	64	39	...	421	

Step 3

Cells are clustered based on FPKM and the estimated AR

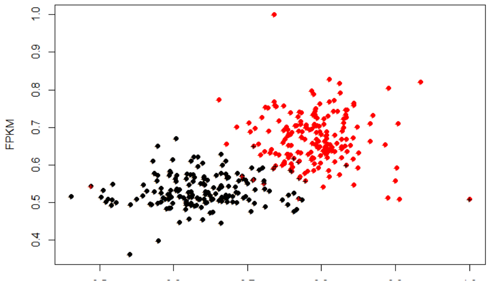

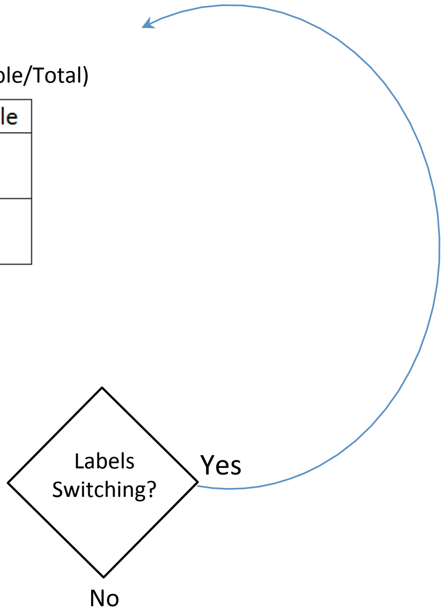

B

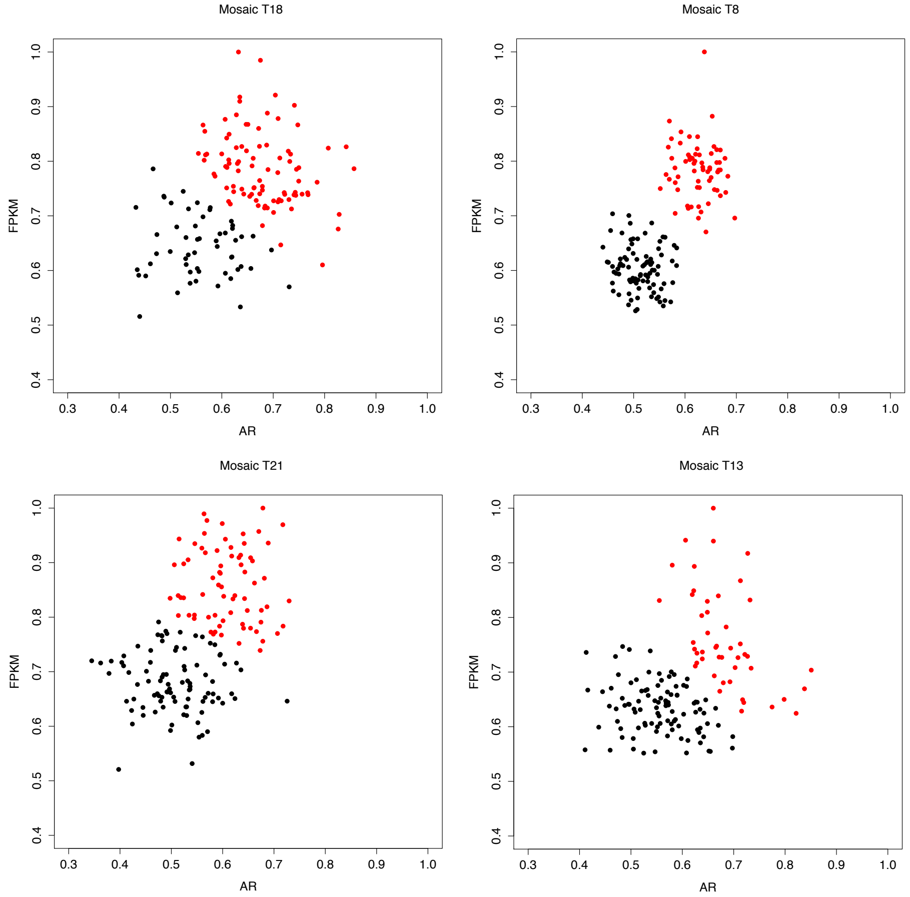

C

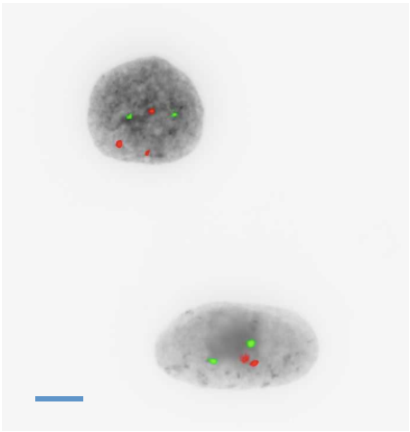

Supplementary Figure 4: **Classification of Euploid and Trisomic cells in mosaic cell populations based on ASE and expression profile of genes located on the supernumerary chromosomes**. A) Classification strategy: Initially cells are clustered with respect to their expression. Putative trisomic cells are then used in order to estimate the genotype of the double allele for all heterozygous sites of the supernumerary chromosome in order to recalculate the Allelic Ratio (AR). K(=2)-means clustering labels the cells as trisomic or euploid. The procedure is repeated until convergence is reached (i.e. no more label switching). Red or black cross indicate wrongly classified cells. B) For all trisomies, the final clusters substantially reflected the levels of mosaicism detected with standard DNA FISH analysis: T18 – Prediction (Fraction of trisomic cells): 60.5% FISH Measured: 57.8%; T21 – Prediction: 44.1% FISH measured: 40%; T8 – Prediction: 43.4% FISH measured: 55.5%; T13: Prediction: 32% FISH measured 34%. C) example of Fluorescent in situ Hybridization (FISH) readout in two neighbouring cells from the mosaic T21 patient (chr21 (red): Vysis, D21S342/D21S341/D21S259 contig probes; chr13 (green - control): Vysis, RB1;13q14 locus). Scale bar: 5µm (see text for details).

Supplementary Figure 5

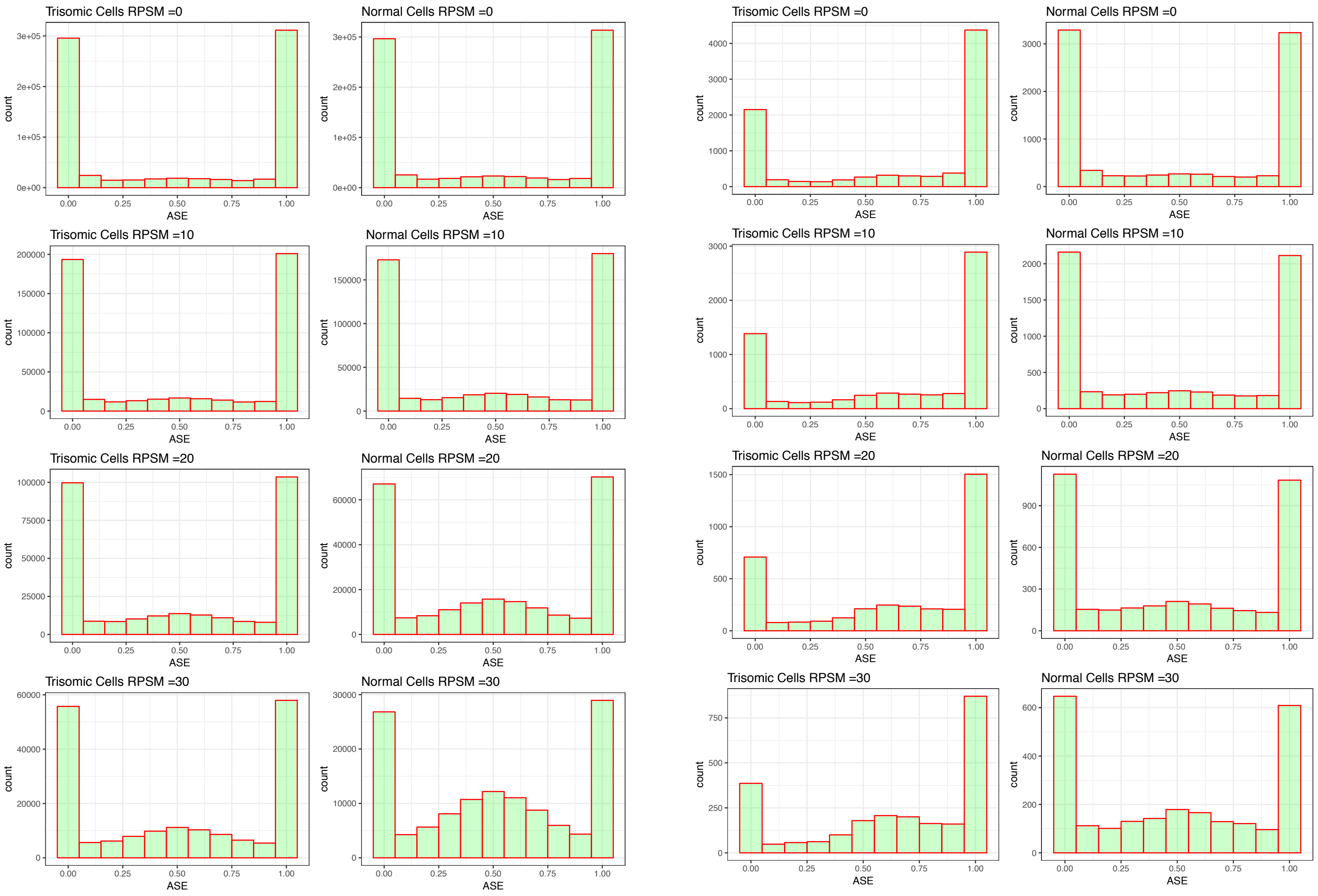

Supplementary Figure 5: Left) Per site whole genome (without Chr21) ASE distribution in trisomic and euploid single cells at RPSM = 0,10,20,30. Right) Chr21 ASE distribution in trisomic and euploid single cells at RPSM = 0,10,20,30. The shape of the distributions is maintained.

Supplementary Figure 6

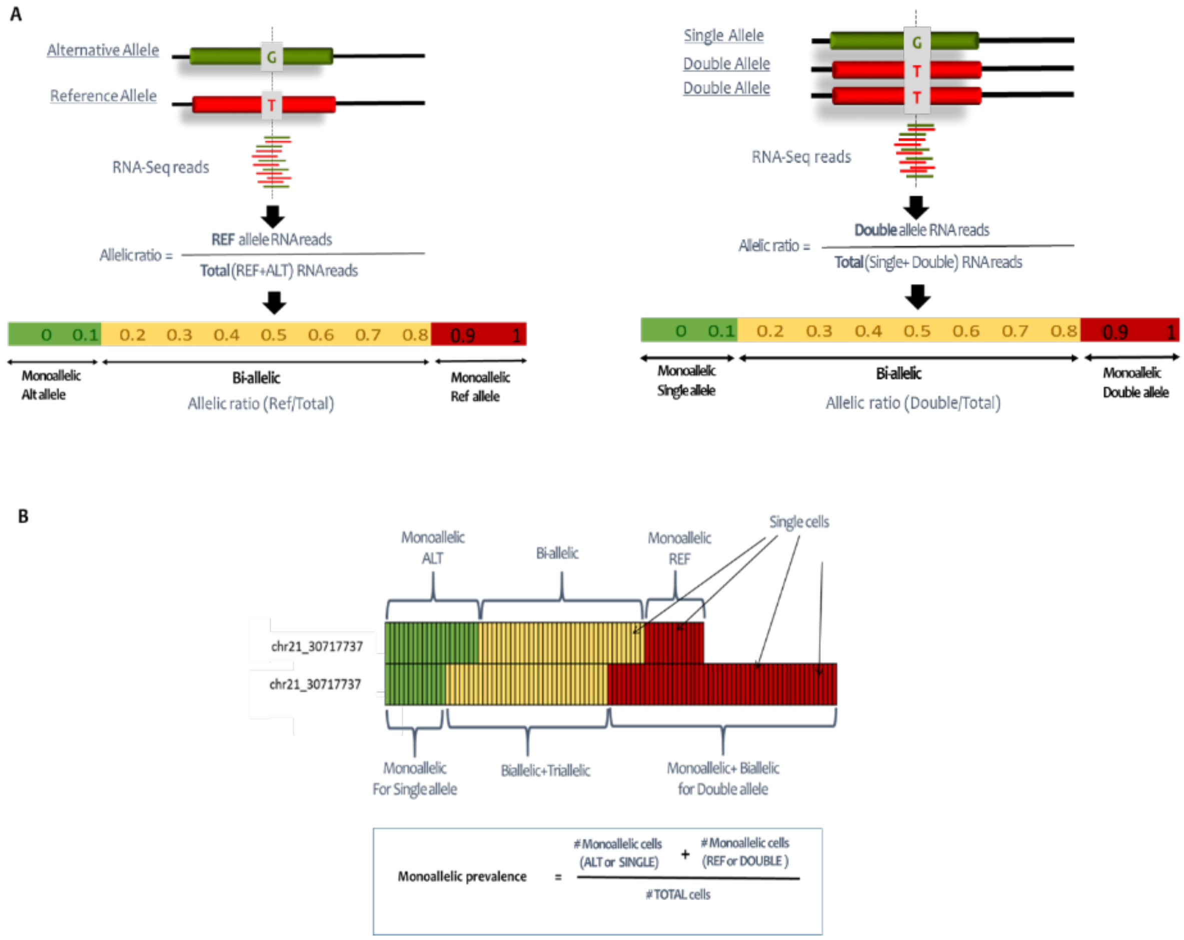

Supplementary Figure 6: A) Left: ASE estimation on the euploid fraction of the genome. Right: ASE estimation on the triploid fraction of the genome. B) Single cell stratification according to monoallelic or biallelic expression. An example of one heterozygous site is illustrated. Upper row: euploid single cell ASE observations; lower row: trisomic single cell ASE observations. Monoallelic prevalence is estimated by calculating the fraction of cells presenting with a monoallelic ASE pattern divided by the total number of expressing cells.

Supplementary Figure 7

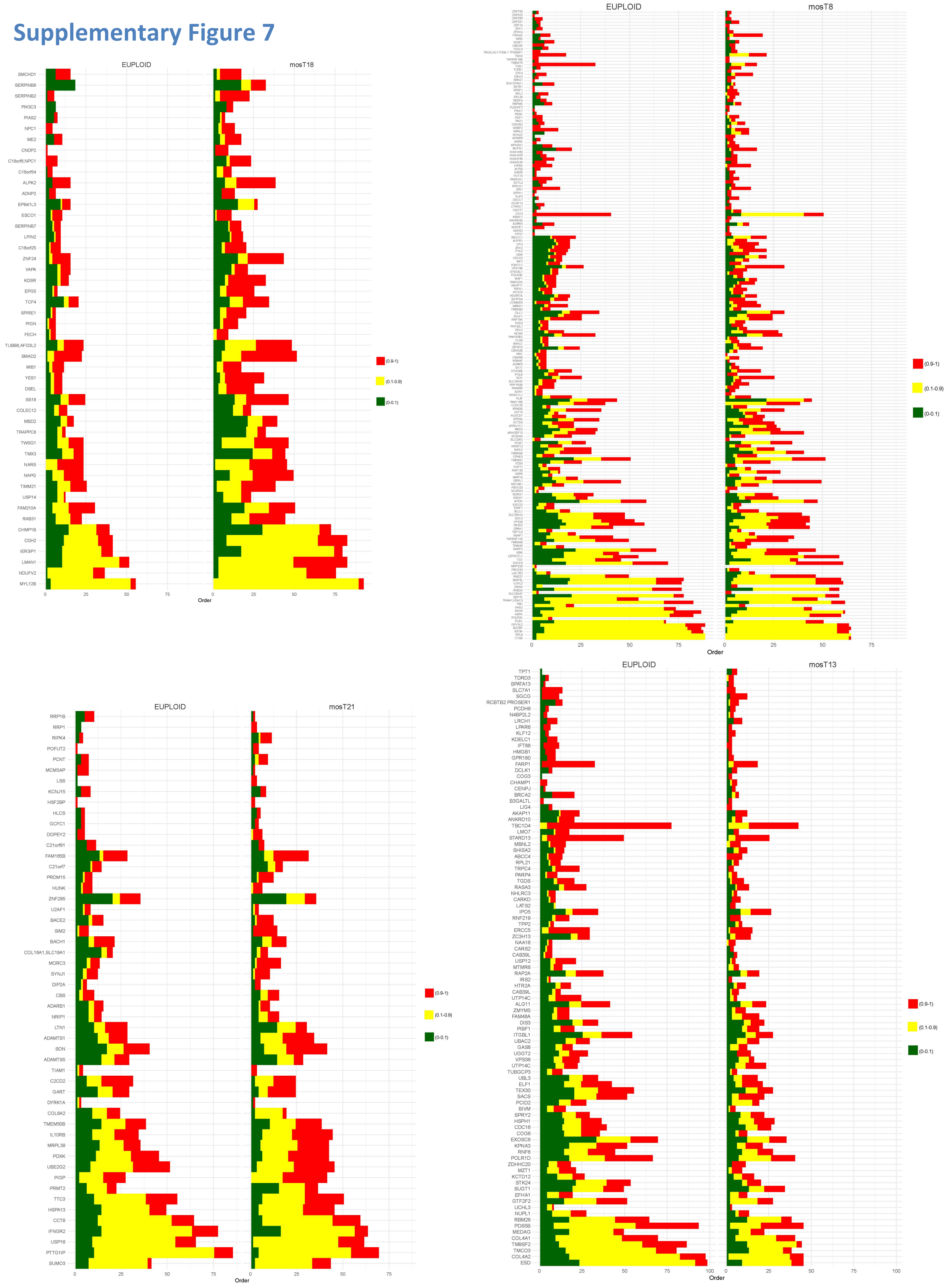

Supplementary Figure 7: Prevalence of monoallelic expression of genes in supernumerary chromosome for the mosaic individuals and respective trisomies.

Supplementary Figure 8

A

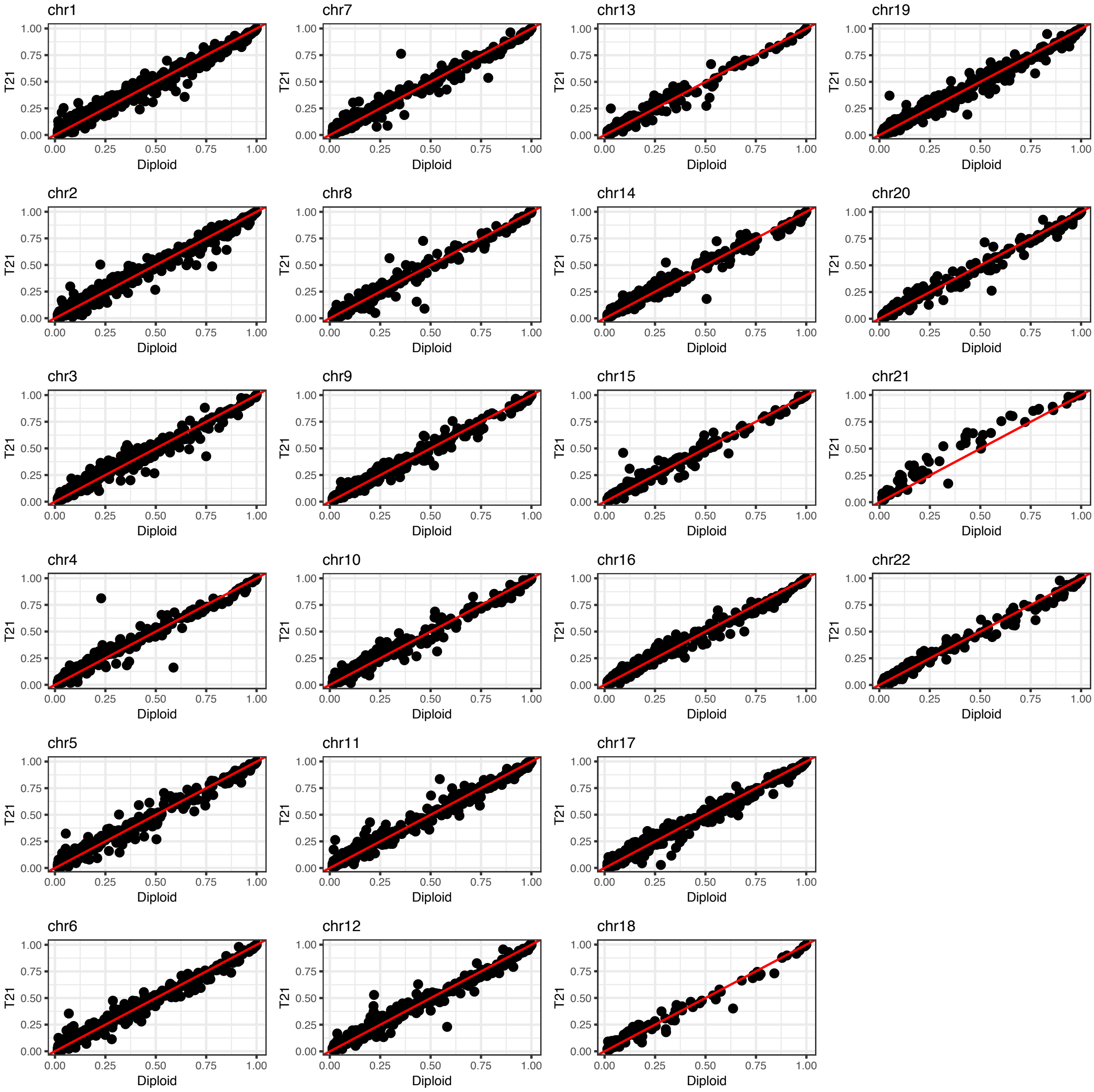

B

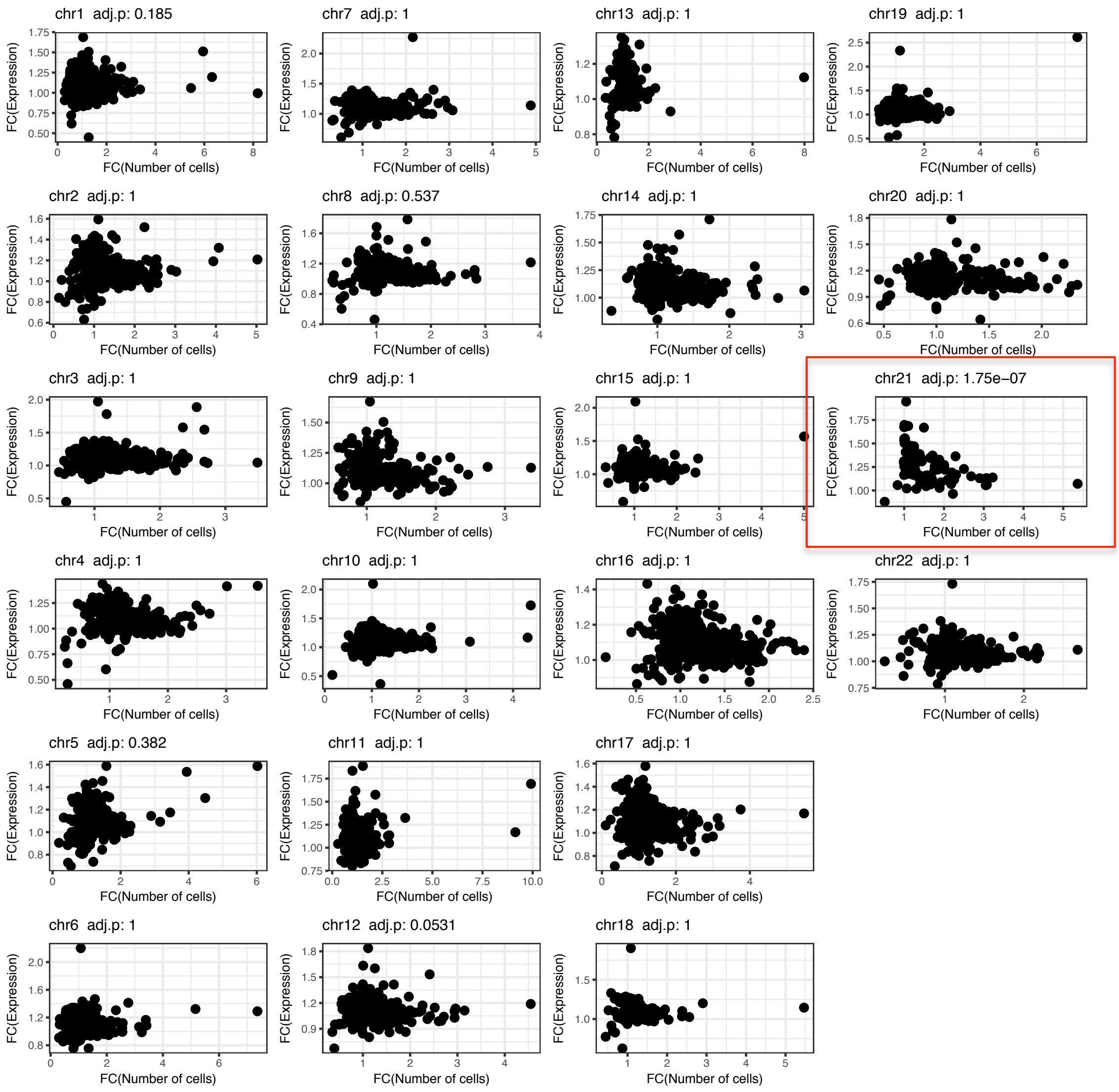

Supplementary Figure 8 : A) (y-axis) whole genome distribution of fraction of trisomic cells expressing genes of each chromosome; (x-axis) distribution of fraction of euploid single cells expressing genes of each chromosome. B) T/D ratio of number of expressing cells vs T/D ratio of single cell expression of genes in all chromosomes and related fitness. Cells with >5 reads and genes expressed in>50 cells have been considered. Fitness to the hyperbolic model is calculated with Spearman correlation. Beside chr21, no other correlation is significant indicating that the hyperbolic model described in equation (1) does not fit to euploid chromosomes.

Supplementary Table 1

Single cell Technology	Samples	Euploid	Trisomy	Total
C1	Discordant Monozygotic Twins for T21	146	170	316
C1	mosT21	95	71	166
C1	mosT18	55	92	147
C1	mosT8	89	64	153
C1	mosT13	99	47	146
10X Genomics	Discordant Monozygotic Twins for T21	3801	4939	8740
	TOTAL	4285	5383	9668

Supplementary Table 1: Samples and single-cell technologies used in this study. In total we investigated 9668 single cell fibroblasts with two different single cell technologies.

Supplementary Note 1

Theoretical Consideration

It has been largely demonstrated that RNA transcriptional activity is of stochastic nature and occurs in bursts with random sizes and frequencies [1–4]. According to the model defined by Raj et al. [1] here we consider the reaction scheme of a two-state promoter:

$$A \xrightarrow{\lambda} I$$
$$I \xrightarrow{\gamma} A$$
$$A \xrightarrow{\mu'} A + m$$
$$m \xrightarrow{\delta} \emptyset$$

where λ is the rate of gene activation (burst frequency), γ is the rate of gene inactivation, μ' is the rate of transcription when the gene is in the active state, and δ is the rate of mRNA decay. m is the number of mRNA molecules. As derived in [1], the probability of observing m mRNA molecules of a low transcribed gene is the steady state solution of following the master equation where the decay rate $\delta << \gamma$ (i.e. each observed read is produced in a new burst):

$$P(m) = (1+\mu)^{-\lambda} \frac{\Gamma(\lambda+m)}{\Gamma(\lambda)\Gamma(m+1)} \left(\frac{\mu}{1+\mu}\right)^{k} \quad (1)$$

It is a Negative Binomial distribution with shape λ and mean μ where we set $\mu = \frac{\mu'}{\gamma}$ as the average number of mRNA molecules produced during the burst. The

expression of the gene g in a bulk of N cells is therefore RV distributed as a sum of N negative binomials. It can be shown that

$$S = \sum_N NB(\mu, \lambda) = NB(N\mu, \lambda). \quad (2)$$

Thus the bulk expression of g is NB distributed with mean $N\mu$ and variance λ. From equation (1), the probability of a single cell to express zero molecules of the gene g is

$$P(0) = (1 + \mu)^{-\lambda}. \quad (3)$$

Accordingly 1-P(0) is the fraction of cells expressing g. Multiplying and dividing (1) by 1-P(0), i.e. the probability to express the gene g in a single cell, gives an equivalent way to write (2):

$$S = [1 - P(0)]NB\left(N\frac{\mu}{1 - P(0)}, \lambda\right). \quad (4)$$

,

where we recognise the last term as the sum of N zero truncated NB (ZTNB) distributions with mean $\frac{\mu}{1-P(0)}$. What we measure in a bulk of cells is the average expression of the gene g, i.e. $E[S(g)]$ across all cells. According to previous studies, the ratio of the expression of g in k copies in a bulk of cells versus an isogenic diploid control is:

$$\frac{E(S_k)}{E(S_2)} = \frac{k}{2}, \quad (5)$$

where $k = 3$ in case of trisomy. Plugging in the mean of equation (2) we obtain:

$$\frac{\mu_k}{\mu_2} = \frac{k}{2}, \quad (6)$$

Plugging equation (4) in equation (5) and replacing equation (3) we obtain:

$$\frac{R_k}{R_2}\frac{\mu_k[1 - (1 + \mu_2)^{-\lambda}]}{\mu_2[1 - (1 + \mu_k)^{-\lambda}]} = \frac{k}{2}, \quad (7)$$

where $R = 1 - P(0)$ is the fraction of cells expressing the gene g. In this form, the left side of the equation (7) can be dissociated in two components: 1) the ratio of the fraction of k-ploid vs diploid expressing cells and 2) the ratio of the average expression of the gene g in a single **expressing** cell. Low expressed genes produce, by definition, a small number of molecules. Using the condition in equation (6) and

taking the limit for $\mu_k \to 0$ of the second component (the ratio of the expressions) of equation (7) we have:

$$\lim_{\mu_k \to 0} \frac{\mu_k [1 - (1 + 2\frac{\mu_k}{k})^{-\lambda}]}{2\frac{\mu_k}{k}[1 - (1 + \mu_k)^{-\lambda}]} = 1, \tag{8}$$

Obviously for the first component of equation (7),

$$\lim_{\mu_k \to 0} \frac{R_k}{R_2} = \frac{[1 - (1 + 2\frac{\mu_k}{k})^{-\lambda}]}{[1 - (1 + \mu_k)^{-\lambda}]} = \frac{k}{2}. \tag{9}$$

The interpretation of these results, obtained by the hyperbolic equation (7) is that low expressed genes tend to be expressed by an increased proportion of cells in tissues where they are present in k copies with respect to normal diploid tissues while maintaining a similar amount of mRNA in each expressing single cell.

Supplementary References

[1] A. Raj, C. Peskin, D. Tranchina, D. Vargas, and S. Tyagi, "Stochastic mRNA Synthesis in Mammalian Cells," *PLoS Biology*, vol. 4, pp. e309+, Oct. 2006.

[2] A. Sanchez and J. Kondev, "Transcriptional control of noise in gene expression," *Proceedings of the National Academy of Sciences*, vol. 105, no. 13, pp. 5081–5086, 2008.

[3] J. R. Chubb, T. Trcek, S. M. Shenoy, and R. H. Singer, "Transcriptional pulsing of a developmental gene," *Current Biology*, vol. 16, no. 10, pp. 1018–1025, 2006. Exported from https://app.dimensions.ai on 2019/01/06.

[4] D. M. Suter, N. Molina, D. Gatfield, K. Schneider, U. Schibler, and F. Naef, "Mammalian genes are transcribed with widely different bursting kinetics," *Science*, vol. 332, no. 6028, pp. 472–474, 2011.
